# Investigation of selected physico-chemical quality parameters in industrial wastewater by electrocoagulation process, Ethiopia

**DOI:** 10.1186/s13065-022-00865-3

**Published:** 2022-09-15

**Authors:** Dessie Tibebe, Almaz Negash, Marye Mulugeta, Yezbie Kassa, Zerubabel Moges, Dereje Yenealem

**Affiliations:** 1 Department of Chemistry, College of Natural and Computational Sciences, University of Gondar, P.O. Box 196, Gondar, Ethiopia; 2 Department of Chemistry, Debre Tabor University, Debre Tabor, Ethiopia

**Keywords:** Electrocoagulation, Physico-chemical quality inductor, Textile effluent

## Abstract

Nowadays, there are more than fourteen major state and private owned textile industries and garment factories in Ethiopia. However, these textile effluents are directly discharged without treatment to the surrounding environment, as a result, the pollutants bring serious problem to the surrounding community including health such as skin diseases, asthma, abortion, carcinogenic effect, biodiversity loss and mutagenic effect on the. The main objective of this study is characterization and treatment of the textile effluent using aluminum electrodes in the electrocoagulation process. EC experimental setups were designed and different parameters were optimized. Electrocoagulation treatment process eliminates physicochemical quality indicators such as pH, electrical conductivity (EC); turbidity, biological oxygen demand (BOD), ammonia; nitrate, nitrite, total nitrogen (TN) and phosphate were determined using standard procedures. From the result, the maximum removal efficiency of phosphate, ammonia, TN, electrical conductivity, turbidity and BOD were obtained 97, 87, 88, 89, 99 and 66%, respectively. Analyses of the electrochemically generated sludge by X-ray Diffraction, Scanning Electron Microscope (SEM), and Fourier Transform Infrared Spectroscopy (FTIR) revealed that the expected crystalline aluminum oxides (bayerite (Al(OH)_3_ diaspore (AlO(OH)) were found in the sludge. The amorphous phase was also found in the floc. Therefore, a treatment technology was good and encourages the community to apply the technique for the treatment of textile effluent before discharging into the environment.

## Introduction

Textile industry has generated wastewater by different production steps. Improper treatment of textile wastewater has released hazardous nature, chemicals and potential to contaminate the environment and can reach to human being through food web. The major physico-chemical quality indicators in the textile effluent are high pH, high temperature, oil, suspended and dissolved solids, leveling agents, toxic and non-biodegradable matter, color, BOD, COD, alkalinity, chlorides, phosphates, nitrates also toxic organic pollutants, and heavy metals [1–5].

Improper and directly discharged without treatment wastewater has widely distributed and difficult for degradation. This causes pollutions to the underground and surface water, land, aquatic ecological system, deficiency of dissolved oxygen, carcinogenic effect, toxicity effect, biodiversity loss and mutagenic effect [6, 7].

Several techniques are used for the treatment of textile effluents including physiochemical methods such as filtration, chemical coagulation, activated carbon adsorption, ultrafiltration, and ozonation [8]. Some of these methods are effective, quite expensive, disadvantages and limitations [9]. In recent years, the successful electrocoagulation (EC) treatment of various organic and inorganic effluents has been reported by many authors[10]. The EC process provides a simple and easily available equipment, easy operation, reliable, cost-effective and environmental friendly method for the treatment of wastewater industrial effluents. It destabilizes of small colloidal particles and eliminating of some coagulants which are hazardous chemicals [5, 11].

Furthermore, the technology is a highly developed and inexpensive water treatment process that has been revealed to be efficient in removing potential metals, organic, inorganic pollutants and breaking down emulsifiers. The kind of coagulant formed will be determined according to the electrode materials used. The electrodes that are commonly used for electrocoagulation process are aluminum plate. This coagulant effects on the coagulation and the efficiency processes. The main reactions take place at the anode electrode [12, 13].

### Aluminum electrode

Anode: Anode: $$Al \to A{l^{3 + }} + 3e$$  

Cathode: $$3{H_2}O + 3e \to {\raise0.7ex\hbox{$3$} \!\mathord{\left/ {\vphantom {3 2}}\right.\kern-\nulldelimiterspace}\!\lower0.7ex\hbox{$2$}}{H_2} + 3O{H^ - }$$

Overall: $$A{l^{3 + }} + 3{H_2}{O_2} \to {\raise0.7ex\hbox{$3$} \!\mathord{\left/ {\vphantom {3 2}}\right.\kern-\nulldelimiterspace}\!\lower0.7ex\hbox{$2$}}{H_2} + Al{\left( {OH} \right)_3}$$

The electrocoagulation technique uses an electrochemical cell to treat the water. In the simplest form, an electrochemical cell consists of two electrodes, the anode and the cathode, immersed in a conducting solution or the electrolyte and connected together via an electrical circuit which includes a current source and control device [12, 13].

In Ethiopia textile industries are playing an important role in economic development and creating job opportunities. There are more than fourteen major state and private owned textile and garment factories. However, the textile effluents are one of the most sources of environmental pollution. These chemicals are directly discharged without treatment, these pollution bring serious problem to the ecological environment such as skin diseases, asthma, abortion, biodiversity loss and mutagenic effect on the surrounding community [6, 7].

The significance of the study is very important to reduce the pollution problem with textile industries in Ethiopia. Therefore, in this study, treatment performance evaluation of textile industrial effluents in the Amhara region, Ethiopia, was investigated by electrocoagulation technique. Generally, the particular impacts of point source studies like industrial effluents in the country in particular to Amhara region are not assessed. Thus it is of paramount importance to evaluate the impact of industrial effluents and their treatment technology to solve community problems living around the industries. Therefore, the main objective of this study is the treatment and characterization of textile effluents using aluminum electrode in the electrocoagulation process.

## Materials and methods

### Study area description and sample collection procedures

**Fig. 1:**
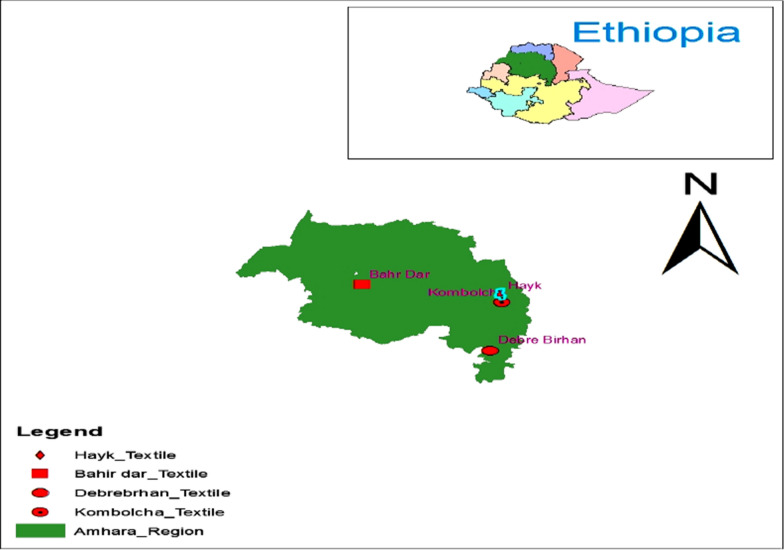
Location of sampling sites

The study was conducted at Bahir Dar, Kombolcha and Debre Brihan Textile industries in the Amhara regional State, Ethiopia. The textile industries effluent samples were collected from Bahir Dar, and Debre Birhan and Kombolcha textile industries effluents in Amhara national regional state (Fig. 1). Bahir Dar and Kombolcha textile effluents were collected after the treatment plant. But Debre Brihan textile industry was collected directly from industrial effluent. The sampling sites were selected based on access, safety, potential sources of pollutions, and wastewater effluents. Corresponding to each site, samples were collected from the out let of textile industries. Prior to sampling, 1L polyethylene bottles was washed and rinsed with distilled water. The samples were collected directly from the industries outlet of the three sampling sites. Samples were transported to University of Gondar and Addis Ababa University for analysis and characterize.

### Apparatus and equipmenf for

The apparatus and equipment’s used in the experiments were portable pH meter (model CT-6021 A), DC power supply regulator (0 − 16 V/0 − 2 A, L 3210, Aplab Limited, India), aluminium plate, magnetic stirrer (30 mm) with hot plate (Remi 5 MLH plus, India), digital balance, oven dry (Macro Scientific Works, India), scanning electron microscope(SE Detector R580, Netherland), XRD (Nicolet 6700, Thermo Scientific, India), and Fourier transform spectrophotometer (Perkin Elmer 65 FTIR, Germany), Portable Spectrophotometer (Wagtech, model 7100, Germany).

### Physico-chemical quality indicators

Physico-chemical Parameters such as Temperature, pH, Conductivity, turbidity, biological oxygen demand (BOD) and dissolved oxygen (DO) were measured in situ with a portable multi meter (HACH MM150)[14]. Ammonia, Nitrate, Nitrite, Phosphate Low Level (LR) and Phosphate High Level (HR) were measured using a Palintest spectrophotometer, 7100. DC power supply regulator (0  –6 V/0–2 A, L 3210, Aplab Limited, India),

### Electrocoagulation setup

The experimental setups for the designed EC process were explained as follows: For each run a 1 L of industrial effluent was mixed with 2 g of sodium chloride which was used as increasing electrical conductivities of the sample and allowed to settle for 30 min and then filtered using Whatman filter paper no 40. The filtered solution was analyzed using spectrophotometer at maximum wavelength of nutrients (nitrate 420 nm, nitrite 520 nm, phosphate 880 nm) to find the residual nutrient concentration. Before and after treatment, the electrodes were washed thoroughly with tap water, and rinsed with distilled water. The pH of the sample was adjusted to a desired value (pH = 7) using (0.5 M) HCl and NaOH solutions and measured by pH meter. In separate two Parallel plates with the same dimension of aluminum electrodes were used in EC technique. External power supply was applied through the Al electrode system (using a DC power source equipped with digital ammeter and voltmeter with a max voltage of 15 V was passed through the industrial effluent via the two electrodes during the 30 min of electrolysis run, respectively. 20 mL of the industrial effluent was taken at different time intervals in each run. The location of the drawn samples was kept constant for each run. The submerged portion of an electrode was 10 × 3 × 1 cm though its actual dimension was 20 × 2 × 2 cm. The distance between the electrodes was kept constant at 2.5 cm and the effective submerged area was 30 cm^2^.

**Fig. 2:**
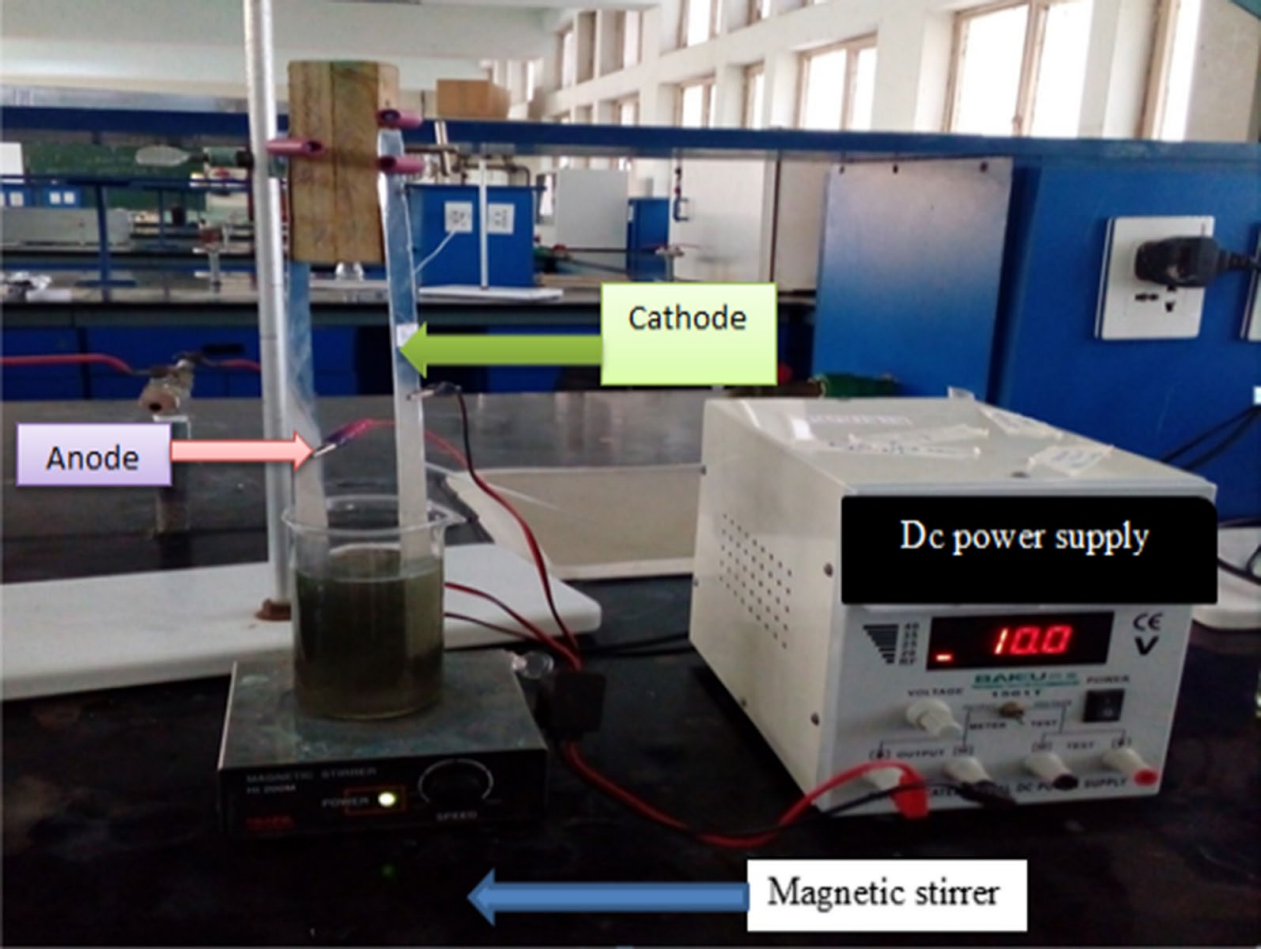
Electrocoagulation setup with two aluminum plate electrodes

The textile effluent in the beaker was continually agitated with a 30 mm magnetic stirrer at 300 rpm during the EC process. Electrodes were rinsed with 5% hydrochloric acid followed by deionized water rinse to avoid the electrode passivation because of the oxidation and contamination of products. All runs were performed at laboratory temperature (25 °C) (Fig. 2). During the experiments, temperature and pH of the industrial effluent was measured by a pH meter (pH meter, CT-6021 A).

The removal efficiency of the selected physico-chemical quality indicators (% Removal) after EC treatment was calculated as follows:

$${\text {Removal\;efficiency}}(\% ) = \frac{{({C_0} - C)}}{{{C_0}}} \times 100$$

Where C_0_ is the initial concentration of selected pollutants before electrocoagulation (mg/L) and C is the final concentration of selected pollutants after electro coagulation(mg/L)[12].

### Characterization of electrodes and sludge

Characterization of aluminum electrodes before and after electrocoagulation process and the sludge products were carried out by Scanning Electron Microscope (SEM), X-Ray Diffraction (XDR), and Fourier Transform Infrared Spectroscopy (FTIR).

## Results and discussion

### Analyses of physico-chemical quality indicators from the textile effluents

The physicochemical quality parameters were investigated before and after treatment of wastewater from the selected textile industries in Amhara regional state (Table 1). All the quality indicators except temperature considerably violate the national standard set by the Department of Environment (DoE) for surface water [15]. The permissible limit of temperature, pH, electrical conductivity, turbidity and DO for discharging into the surface water is 40^°^C, 6 to 9, 1200 µS/cm, 5 NTU and 4.5 to 8 mg/L, respectively.

**Table 1:** The physico-chemical quality indicators measured before and after EC treatment process (electrolysis time = 30 min and pH = 7, max voltage = 15 V) from the three textile effluents

Sites	Bahir Dar (BD)			Debre Brihan (DB)			Kombolcha (KO)		
Water quality parameters	Before EC	After EC	RE (%)	Before EC	After EC	RE (%)	Before EC	After EC	RE (%)
Nitrate (mg/L)	9.24	1.50	84	0.85	0.06	93	2.00	0.97	52
Nitrite (mg/L)	0.624	0.038	94	0.030	0.002	93	0.178	0.08	55
Phosphate HR (mg/L)	1.8	0.9	50	18.0	9.4	48	14.0	6.4	54
Phosphate LR (mg/L)	0.06	0.03	50	3.76	2.00	47	1.25	0.04	97
Ammonia (mg/L)	8.00	1.00	88	20.00	2.60	87	0.63	0.02	61
Total nitrogen (TN) (mg/L)	17.86	2.54	86	20.88	2.66	87	2.81	1.07	62
PH	5.10	7.6	–	5.7	7.5	–	6.5	8	–
Conductivity($\mu$s/cm)	557	113	79	1257	140	88.86	631	102	83.84
Temperature (ºC)	30.38	22.8	–	23.6	22.6	–	27.6	22.8	–
DO (mg/L)	3.74	4.52	–	3.39	4.83	–	4.49	4.81	
Turbidity (NTU)	0.60	0.05	92	42.60	0.43	99	6.10	0.02	99
BOD (mg/L)	4.45	1.54	65	4.69	1.59	66	4.19	1.65	61

Whereas* RE* removal efficiency,* EC* electrocoagulation

#### Temperature

The effluents were measured in-situ during day time between 9 AM and 3 PM. The mean temperatures ranged from 23.6 to 30.38 °C, the highest values were measured at Bahir Dar textile effluent (30.38) while the lowest values were measured at Debre Birhan textile effluent (23.6) (Table 1). Temperature has its own effect on certain chemical and biological reactions takes place in water and in organisms in the aquatic ecosystem which depends upon seasons and time of sampling. Temperature has also an effect on other water quality indicators like DO, pH, EC [13].

#### pH

The pH Values ranged from 5.1 to 6.5 and 7.5 to 8 before and after treatment, respectively (Table 1). According to WHO (2011) [16] and EEPA (2003) [17], pH ranged from 6.5 to 8.5 and 6 to 9 was suitable for aquatic organisms and humans, respectively. Therefore, as the present study shows the textile wastewater pH value is suitable for the surrounding community after treatment by electrocoagulation pH variation of this experiment using Aluminum as a cathode and anode. It was found that Aluminum can exist in several forms based on the pH of the solution. In the acidic zone up to pH 3, cationic species which are soluble were found to be predominant Al^3+^ and Al(OH)_2_^+^, and hence removal was found to be lower. When pH is in the range of 4 to 9, monomeric and polymeric aluminum species are formed. These get converted to Al(OH)_3_ by complex polymerization, which is very useful in the removal process. Therefore, the removal mechanism of pollutants is based on their adsorption on the Al(OH)_3_ flocs. Increases in pollutant removal at higher pH values may be due to the nature of the reaction between Al^+ 3^ ions and hydroxide ions [4, 5].

#### Electrical conductivity (EC)

The electrical conductivity of wastewater from the studied textile industries were 557, 631 and 1257 $\mu$S cm^− 1^ in Bahir Dar, Kombolcha and Debre Birhan industry, respectively (Table 1). After treatment, the conductivity of wastewater was reduced to 79, 88.88 and 83.8% in Bahir Dar, Kombolcha and Debre Birhan industries, respectively. Higher electrical conductivity values were recorded at Debre Birhan textile industry than other industries. Unfortunately, except Debre Birhan textile industry all the measured values were below the maximum permissible limit of 1200 µS cm^− 1^ (DoE, 2003) and1000 µS cm^− 1^ (EEPA, 2003) [17, 18]. Electrical conductivity was directly proportional to the total dissolved solids (TDS) [19]. The reduction of the electrical conductivity in aquatic ecosystem is important to aquatic life and animal species and can survive within a certain ranges [20]. Therefore, from the result, the EC technology is an efficient technique for the reduction of cations and anions in textile effluents

#### Turbidity removal

Turbidity is an important aspect of textile wastewater. Turbidity prevents the penetration of sunlight and oxygen transfer process in water, and as a result it affects the survival of aquatic life. Sample effluents contained higher amount of turbidity than the standard of DoE (5 NTU) and needs treatment to safely discharge in the surface water. By using electrocoagulation method reduction of turbidity becomes effective in the three textile industries. In case of 30 min-15 V, turbidity decreased from 42.6, 0.60 and 6.1 NTU to 0.43, 0.05, and 0.02 NTU for samples Debre Briha, Bahir Dar and Kombolcha respectively. Turbidity removal percentage was achieved from 92 to 99% at 30 min-15 V with an average of 96% removal. This result as shown in Table (1) is supported by Islam et al. [18].

#### Biological oxygen demand (BOD)

BOD decreased from 4.69, 4.45 and 4.19 mg/L to 1.59, 1.54 and 1.65 mg/L for samples of Debre Brihan, Bahir Dar and Kombolcha respectively. The removal efficiencies of BOD in the textile effluents ranged from 61 to 66% after treatment. The higher the concentration of BOD, more the extent of oxygen depletion in the water bodies [21]. According to EEPA (2003), the maximum permissible limit of BOD is 50 mg/L. DO is greatly influenced by the BOD level in wastewater [21]. As a result, wastewater treatment techniques through electrocoagulation methods in textile industries become preferable.

#### Increase dissolved oxygen (DO)

**Fig. 3:**
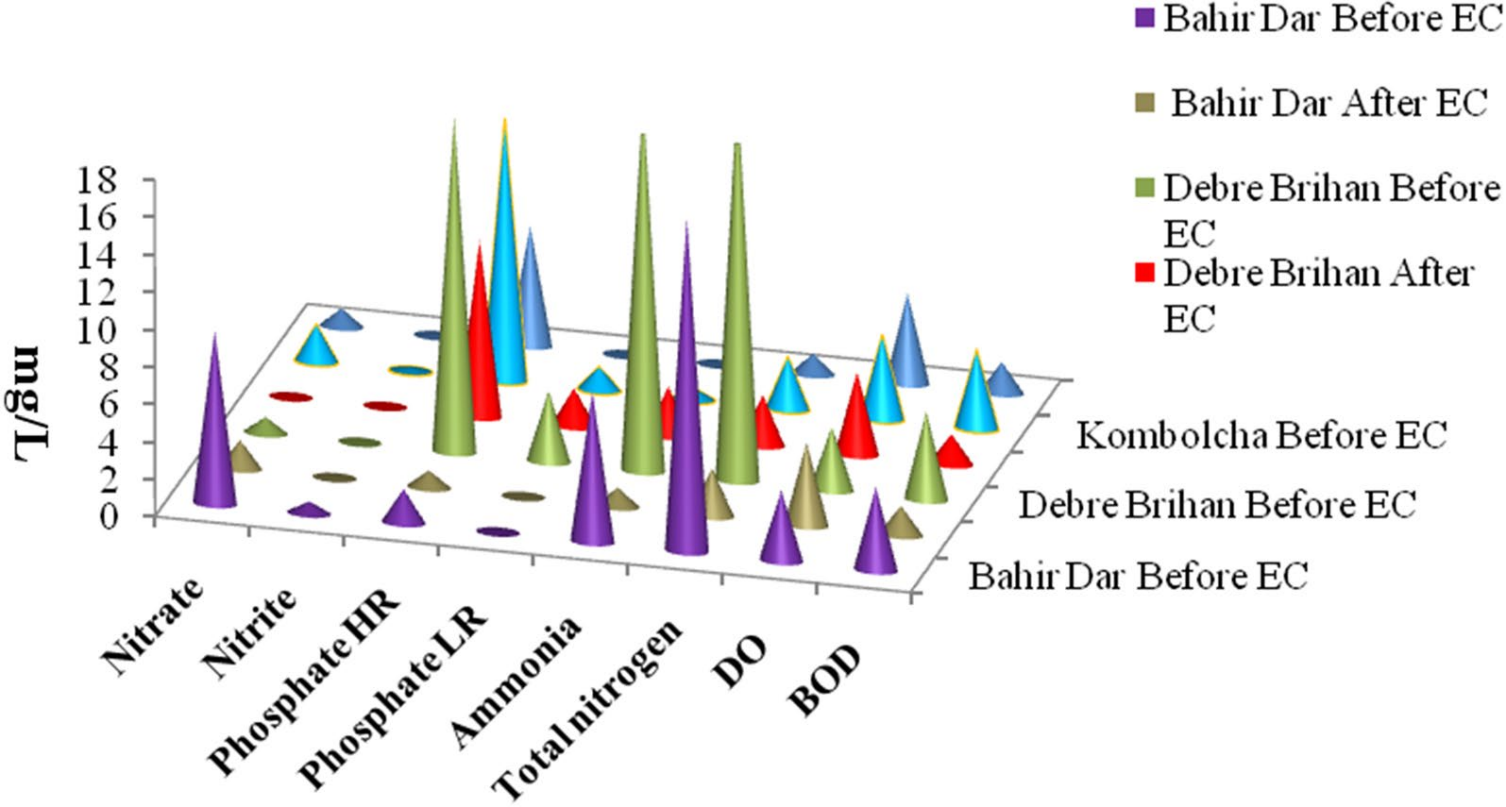
The physico-chemical quality indicators distribution of textile effluents before and after treatment process

DO is essential for the survival of aquatic life, thus it serves as an important indicator of ecosystem condition and dependent on the chemical, physical and biochemical activities occurring in the water. DO concentrations are directly dependent on oxygen generation through photosynthesis and consumption by living organisms [22]. All the samples contained low DO level than DoE standard. For operational condition, DO increase from 3.74, 3.39, and 4.49 mg/l to 4.52, 4.83, and 4.81 mg/l for samples Bahir Dar, Debre Birhan and Kombolcha textile industry respectively (Table 1; Fig. 3). Therefore; as the result shows the treatment of textile wastewater by aluminum electrocoagulation method was efficient improvement of the dissolved oxygen level. This result as shown in Table (1) is supported by Islam et al. [18]

#### Phosphates

On treatment, the phosphate LR in the wastewater samples from all the under studied textile industries were reduced to 0.03, 2.00 and 0.04 mg/L and the removal efficiencies were 50%, 47%, and 97% in Bahir Dar, and Debre Birhan and Kombolcha textile industries, respectively (Table 1; Fig. 3).The phosphate HR level of wastewater from Bahir Dar, Debre Birhan and Kombolcha textile industries were 1.8, 18.0, and 14.0 mg/L and reduced to 0.9, 9.4 and 6.4 mg/L after treatment, respectively. The removal efficiencies were 50%, 48%, and 54% in Bahir Dar, and Debre Birhan and Kombolcha textile industries respectively. Excessive presence of phosphate in conjunction with nitrates causes algal blooms which result in the death of aquatic organisms [21]. According to EEPA (2003) increasing of phosphate above maximum permissible limit (1 mg/L) allowed to the growth of aquatic plants which enhance the shortage of DO [17].

#### Nitrate, nitrite and ammonia

The nitrate concentration from Bahir Dar, Debre Birhan and Kombolcha textile industries were 9.24, 0.85, and 2.00 mg/L and reduced to 1.50, 0.06 and 0.97 mg/L before and after treatment, respectively. The concentrations of nitrite, ammonia and total nitrogen in all sites were significantly decreased after treatment (Table 1; Fig. 3). The concentration of ammonia was higher before treatment this leads to organic N is converted to the inorganic nitrogen pool through bacterial decomposition and excretion of NH_4_^+^ and amino acids by living organisms. According to EEPA (2003) the maximum permissible limit of ammonia is 30 mg/L, beyond which most aquatic organisms affected [17]. Therefore, electro coagulation process is an effective and recommended technique for the treatment and characterization of textile effluents.

### Results of Spectroscopic and microscopic techniques

#### Results for SEM analyses

**Fig. 4:**
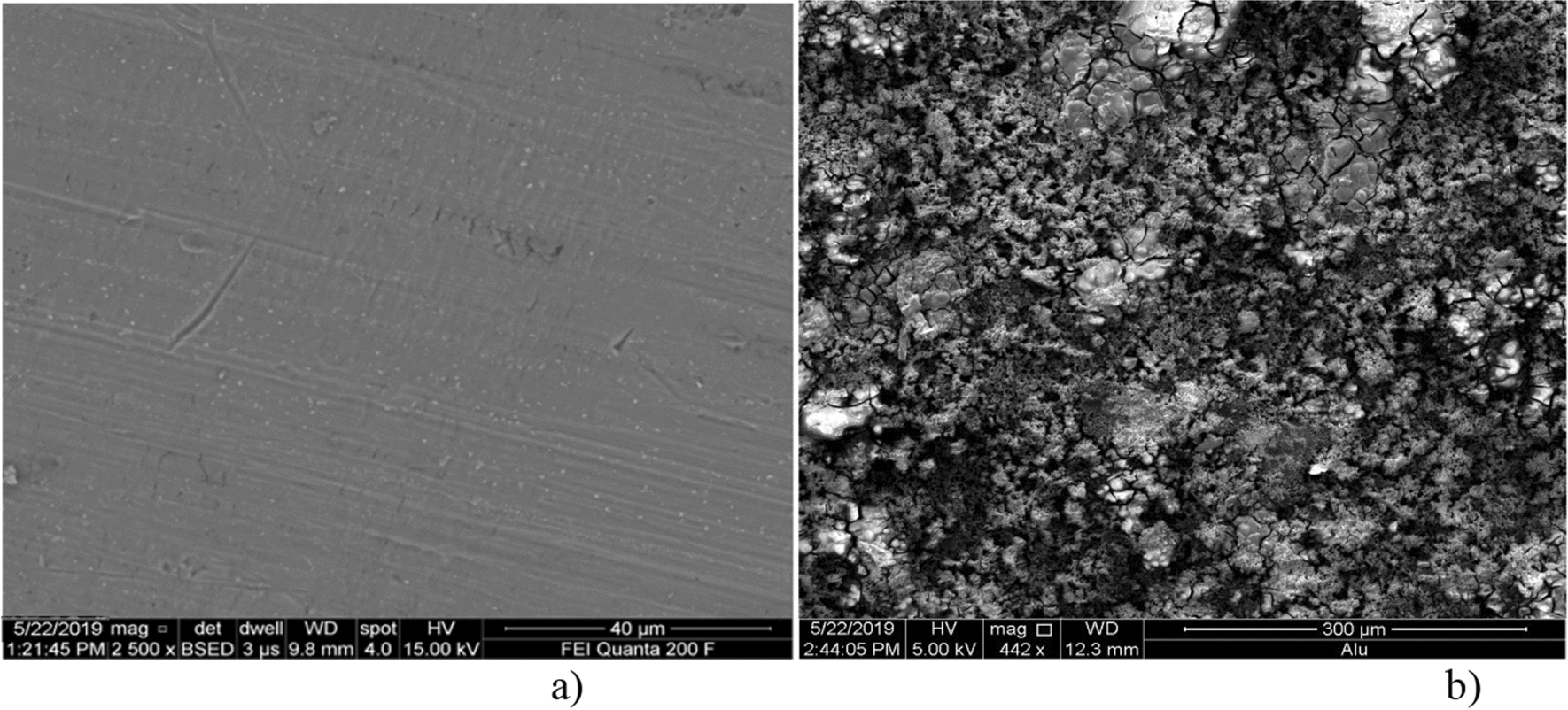
SEM micrography of typical surface morphology of **a** aluminum plate electrode before treatment, **b** aluminum anode electrode after electrolysis

SEM images of the two electrodes before and after EC were obtained to compare the surface morphology. As shows in Fig. 4a, the original aluminum plate surface prior to its use in EC; the surface of the electrode is uniform. From the results of SEM analyses, the same electrode after use in electrocoagulation becomes morphological change (Fig. 4b).

**Fig. 5:**
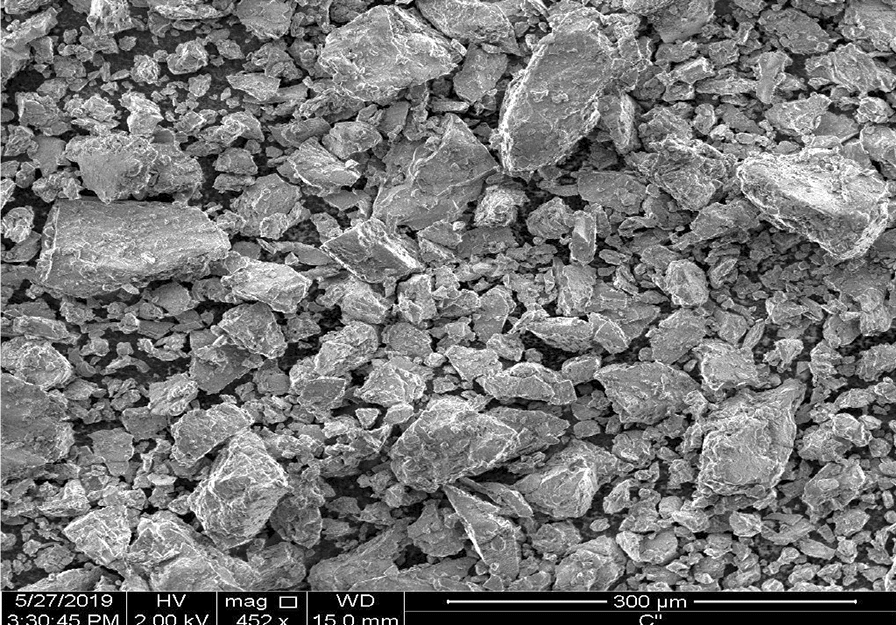
SEM micrography of residue after electrolysis

The electrode surface brings into being to be uneven with a number of holes. These holes are formed around the nucleus of the active sites where the electrode dissolution takes place to produce the Al (OH) _3_. The creation of great number of hole probably attributed to the of anode material utilization at active sites due to the generation of oxygen at its surface. Adsorbed species from the solution on to the active sites of pores and onto the flocs and the residue particles also show higher resistance to degradation [23]. The residues reveal that, overall appearances of the treated product (Fig. 5). The particles are rectangular with different sizes. It can be observed that product aggregation is constituted by various irregular particles with a variety of pores and voids due to the evolution of large quantity of gases [23].

#### Results of XRD analyses

**Fig. 6:**
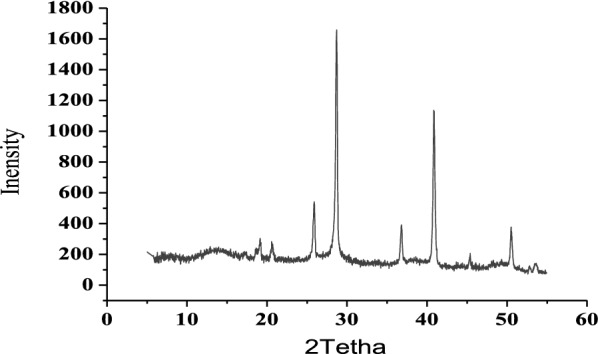
XRD diffractograms of the sludge obtained after electrolysis (unit of intensity (a.u) & 2Tetha (degree))

Figure 6 showed the sharp diffraction peaks in the spectrum of Al–Al electrode sludge. Bragg reflections possessing very sharp peaks and high intensity indicate that the analyzed phase possesses are a long-range order, i.e., amorphous or poorly crystalline. Most Al hydroxides are found to be very poorly crystalline, due to these properties; the formation of crystallization structure is a very slow process during the electrocoagulation process. However, the previous literature on the amorphous nature of the aluminum oxide layer supported this result by reporting that the oxide film does not contain a pure crystalline aluminum compound, but contains an amorphous aluminum compound [24].

According to research on barrier-type films, the alumina in the film has been accounted as $\gamma$ alumina. The $\gamma -$alumina has possessions that lie between amorphous alumina and crystalline alumina [25].

#### FTIR analysis of sludge

**Fig. 7:**
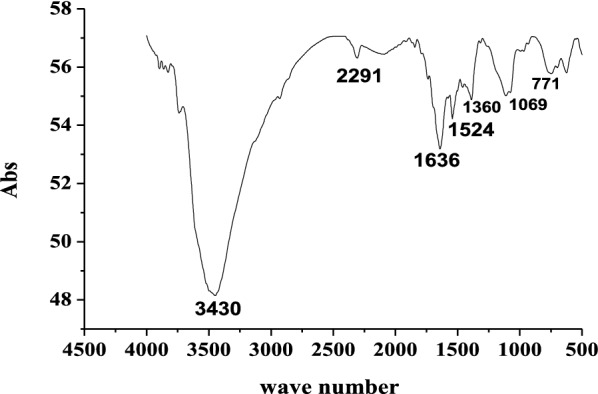
FTIR spectra for the sludge obtained after electrolysis (wave number = cm^− 1^)

FTIR spectra of the sludge obtained after electrocoagulation process is shown in Fig. 7. The typical feature of the FTIR spectrum of the sludge varies depending on the functional group of the obtained compound.

The large wide peak at 3430 cm^− 1^ is due to the O–H stretching vibration. C-H stretching of methyl groups appears at around 2291 cm^− 1^. The peak at 1524 cm^− 1^ was attributed to the stretching vibration of the C=C and C=N aromatic groups and at 1636 cm^− 1^ attributed to hydroxyl bending. The peak at 1069 cm^− 1^ indicates C-O stretching, and the peak at 771 indicates Al–O–H bending. Therefore, FT-IR analysis of the sludge of Al–Al electrodes suggested the presence of hydroxyl groups. Basic hydroxyl group and corresponding OH stretching was identified at 3430 cm^− 1^ for aluminum hydroxide/oxyhydroxides phases. Similar results have been reported elsewhere [26–29]. From FTIR analysis of the Al electrode by-product chemical speciation of this amorphous phase can be aluminum hydroxide and/or aluminum oxyhydroxides.

## Conclusions and recommendations

Good results in the treatments and characterization of industrial waste water by electrochemical techniques for the removal of physicochemical quality indicators were obtained. The results of SEM showed the various morphologies of the electrodes before and after treatments as well as the sludge product. The SEM result demonstrated very fine structures for aluminum hydroxide. From XRD and, FTIR analyses of the residue, one can conclude that the chemical speciation of the by-products can be mostly aluminum hydroxide and aluminum phosphate. Therefore, it is better to use the electrocoagulation method for textile effluent treatment before discharging into the environment.

## Data Availability

All data generated and analyzed are included within this research article.

## Declarations

### Sample collection permission

during sample collection our institute gave support letter to give permission for sample collection and the textile industries owners gave permission for sample collection and in situ measurements.
